# Safety of continuing aspirin therapy during spinal surgery: A systematic review and meta-analysis

**DOI:** 10.1097/MD.0000000000008603

**Published:** 2017-11-17

**Authors:** Chenggui Zhang, Guodong Wang, Xiaoyang Liu, Yang Li, Jianmin Sun

**Affiliations:** a Department of Spine, Shandong Provincial Hospital Affiliated to Shandong University, Jinan, Shandong Province; b Anatomical Institute of Minimally Invasive Surgery, Southern Medical University, Guangzhou, China.

**Keywords:** aspirin administration, preoperative period, spinal fusion, spinal surgery

## Abstract

**Background:**

Questions whether to continue or discontinue aspirin administration in the perioperative period of spinal surgery has not been systematically evaluated.

**Objective:**

The present systematic review is carried out to assess the impact of continuing aspirin administration on the bleeding and cardiovascular events in perispinal surgery period.

**Methods:**

Studies were retrieved through MEDLINE, EMBASE, and Springer Link Databases (search terms, *aspirin, continue or discontinue*, and *spinal fusion*), bibliographies of the articles retrieved, and the authors’ reference files. We included studies that enrolled patients who underwent spinal surgery who were anticoagulated with aspirin alone and that reported bleeding or cardiovascular events as an outcome. Study quality was assessed using a validated form. 95% confidence interval (95% CI) was pooled to give summary estimates of bleeding and cardiovascular risk.

**Results:**

We identified 4 studies assessing bleeding risk associated with aspirin continuation or cardiovascular risk with aspirin discontinuation during spinal surgery. The continuation of aspirin will not increase the risk of blood loss during the spinal surgery (95% CI, −111.72 to −0.59; *P* = .05). Also, there was no observed increase in the operative time (95% CI, −33.29 to −3.89; *P* = .01) and postoperative blood transfusion (95% CI, 0.00–0.27; *P* = .05). But as for the cardiovascular risk without aspirin continuation and mean hospital length of stay with aspirin continuation, we did not get enough samples to make an accurate decision about their relations with aspirin.

**Conclusion:**

Patients undergoing spinal surgery with continued aspirin administration do not have an increased risk for bleeding. In addition, there is no observed increase in the operation time and postoperative blood transfusion.

## 1 Introduction

In patients who are receiving aspirin therapy and require spinal surgery, some authors hold the opinion that low-dose aspirin should be discontinued at least 7 days before spinal surgery because of the bleeding risk.^[1]^ However, in recent years, some clinical results show that aspirin should not be discontinued for the concomitant thromboembolic risks associated with aspirin withdrawal.^[2]^

Aspirin produces its effect primarily by influencing the biosynthesis of cyclic prostanoids, thromboxane A2 (TXA2), prostacyclin, and other prostaglandins. With the use of aspirin, cyclooxygenase-1 (COX-1) is completely inactivated, whereas COX-2 converts arachidonic acid not to prostaglandin H2 (PGH2), but to 15-R-hydroxyeicosatetraenoic (15-R-HETE). Aspirin-induced inhibition of TXA2 and PGI2 has an effect on anti-hemostasis.^[3,4]^ Aspirin, by virtue of its ability to inhibit platelet aggregation and prevent thrombosis, has been widely used in the secondary (preventing recurrence of disease) and primary (preventing first occurrence of disease) prevention of acute myocardial infarction and stroke.^[1]^ Low-dose aspirin is now commonly used for preventive purposes in patients who have had myocardial infarction and stroke.^[5]^

Aspirin blocks cyclooxygenase and inhibits the aggregation of platelets. However, the impaired function of platelets is irreversible during their life span. On the contrary, the antiplatelet action of aspirin exerts an undesirable effect that increases the risk of hemorrhage during surgery. Despite evidence to the benefit of antiplatelet therapy in patients at risk of cardiac and cerebrovascular complications, aspirin treatment is often discontinued before surgery because of the risk of perioperative bleeding.^[6,7]^ Because of the characteristic that aspirin impairs platelet aggregation, a study named “Antithrombotic Effects of Aspirin on 1- or 2-Level Lumbar Spinal Fusion Surgery”, which contains 182 samples show that to reduce the intraoperative blood loss and the postoperative blood transfusion, stopping the use of aspirin 7 to 10 days before spinal surgery is necessary.^[7]^

To our knowledge, there are no accurate reports on the effect of aspirin on spine surgery. Some studies demonstrated that aspirin could be a risk factor for epidural hematoma. Blood loss during surgery is one of the major concerns for spine surgeons.^[8]^ However, some clinical results show that aspirin should not be discontinued for the thromboembolic risks.^[1,2]^ We, therefore, undertook a systematic review of the literatures relating to continuation of aspirin around the time of spinal surgery. Our primary objective was to assess the safety (bleeding risk) of continuing aspirin therapy during spinal surgery. We also evaluated the cardiovascular risk with perioperative aspirin continuation. At the same time, operative time, postoperative blood transfusion, and mean hospital length of stay were also included to assess the influence of the continuation of aspirin.

## 2 Methods

### 2.1 Selections of studies

All the databases (MEDLINE, EMBASE, and Springer Link) were searched with aspirin, continue or discontinue, and spinal fusion as keywords. In addition, a manual search was performed using the authors’ reference files and reference lists from original communications and review articles. After literature search, 80 studies were identified, which were reviewed to determine whether they met the criteria for inclusion. This was a meta-analysis based on previous published studies, thus no ethical approval and patient consent were required.

The literature search and article review were conducted independently by 2 reviewers (CZ, XL) with disagreements resolved by consensus. Studies were included if they met the following criteria: an original article, patients were anticoagulated with aspirin throughout spinal surgery, and reported bleeding events associated with the procedure. Studies were excluded if patients were anticoagulated with a medication other than aspirin (or in combination with aspirin). Studies on nonspinal surgery were also excluded.

### 2.2 Study quality assessment

Quality assessment was performed independently by 2 reviewers (CZ, YL) using the validated Newcastle–Ottawa scale (NOS) for cohort studies. The NOS for cohort studies was also used to evaluate the quality of case series, as it includes several items related to a control group, so the lower quality of the case series would be reflected in lower scores on these items. Disagreements were resolved through consensus.

### 2.3 Data abstraction

Data abstraction was performed independently by 2 reviewers (CZ, YL) with disagreement resolved through consensus. The reviewers were not blinded to study name or author.

### 2.4 Statistical analyses

Statistical analyses were performed using meta-analysis software package (Review Manager 5.3; Cochrane Collaboration, Oxford, UK). The data of 4 studies were calculated to be analyzed by the RevMan 5.3 statistical software package (Cochrane Collaboration, Oxford, UK). Proportions were converted to a natural logarithmic scale using Microsoft Excel 2013 (Microsoft office, Microsoft). If no events occurred, a correction of 0.5 was added in order to obtain a natural logarithm for that proportion. However, this is represented in the forest plot as an event rate of 0.01. The delta method was used to calculate standard errors for proportions. Statistical pooling of 95% confidence interval (95% CI) was done using RevMan 5.3. Statistical heterogeneity beyond chance was evaluated using the *I*^2^ value. The more conservative random effects model was used for pooling of the outcomes.

## 3 Results

### 3.1 Literature search

After the duplicate studies were excluded, a total of 80 studies were obtained. On the basis of the title and abstract, 76 reports were excluded because the topic of the article was not relevant to the objective of the review. Finally, 4 studies were included and analyzed.

### 3.2 Study characteristics

All the concrete information about included studies are listed in Table 1. ^[5,7,9,10]^ Three studies have lumbar fusion, while 1 study combined cervical, thoracolumbar, and lumbar therapy. Two studies are anticoagulated with aspirin and the other 2 with ASA (they choose aspirin as the representation of ASA). We collect the data about bleeding events, cardiovascular events, operation time, hospital length of stay, and postoperative blood transfusion from these 4 articles. All studies reported blood loss. Cardiovascular events, hospital length of stay, operation time, and postoperative blood transfusion were recorded in 3 studies.

**Table 1:**
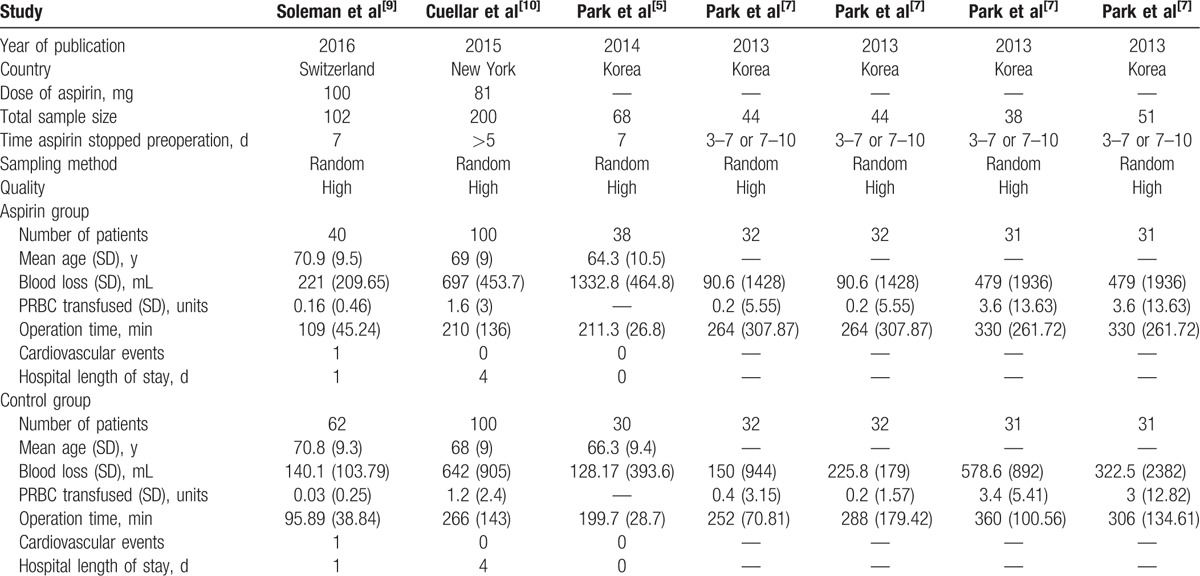
Characteristics of included studies.

### 3.3 Study quality

The 4 cohort studies were of good quality, with 6 to 7 out of all the 8 NOS criteria satisfied. The main limitation of these studies was unequal numbers between the anticoagulated and non-anticoagulated (control) groups. Also, no study met all 8 NOS criteria.

### 3.4 Study outcomes

**Figure 1:**
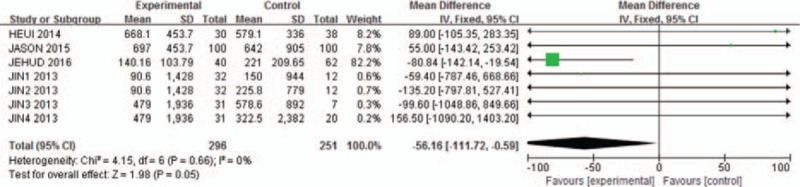
Meta-analysis of included studies comparing estimated blood loss between aspirin group and control group. Significant difference was observed. Continuation of aspirin will not increase the risk of blood loss.

**Figure 2:**
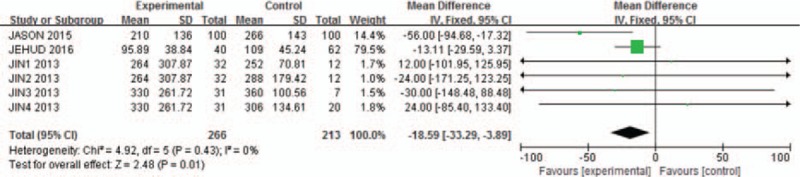
Meta-analysis of included studies comparing operation time between aspirin group and control group. Significant difference was observed. Continuation of aspirin will not increase the operation time.

**Figure 3:**
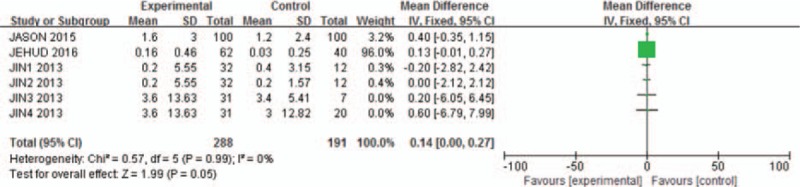
Meta-analysis of included studies comparing postoperative blood transfusion between aspirin group and control group. Significant difference was observed. Continuation of aspirin will not increase the risk of postoperative blood transfusion.

**Figure 4:**
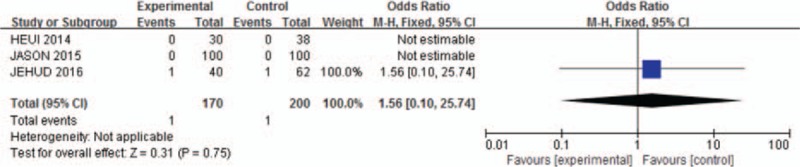
Meta-analysis of included studies comparing cardiovascular events between aspirin group and control group. No significant difference.

**Figure 5:**
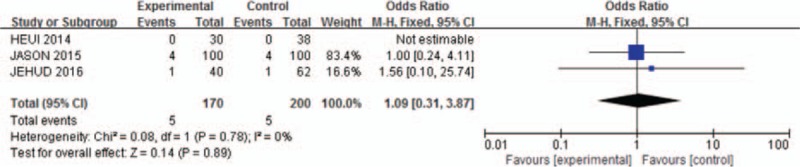
Meta-analysis of included studies comparing hospital length of stay between aspirin group and control group. No significant difference.

Agreement between reviewers on data abstraction was excellent (raw agreement on raw and calculated outcome items = 0.94). As shown in Fig. 1, the pooled incidence of bleeding for all studies was −56.16 (95% CI: −111.72 to −0.59). The continuation of aspirin will not increase the risk of blood loss during the spinal surgery (*P* = .05, *I*^2^ = 0%). As shown in Fig. 2, pooling the results of all the studies suggested that preoperative aspirin did not result in increased operation time (95% CI, −33.29 to −3.89; *P* = .01). As shown in Fig. 3, the pooled incidence of postoperative blood transfusion for all studies was 0.14 (95% CI: 0.00–0.27). Significant difference was observed across studies for this outcome (*P* = .05, *I*^2^ = 0%). As shown in Fig. 4, pooling the results of all the studies showed no significant difference in cardiovascular events between the 2 groups (95% CI: 0.10–25.74, *P* = .75). As shown in Fig. 5, the pooled incidence of hospital length of stay for all studies was 0.88 (95% CI: 0.25–3.18). There was no significant difference (*P* = .85, *I*^2^ = 0%) across all included studies for this outcome.

## 4 Discussion

It has recently been verified safe to perform orthopedic surgery while continuing low-dose aspirin therapy, but none study has been performed on spine surgery.^[11]^ Alghamdi et al^[12]^ report that aspirin therapy before surgery may be associated with the increase of chest tube drainage and requirement for blood products in patients undergoing coronary artery bypass grafting surgery. Similarly, Merriman et al^[13]^ hold the opinion that the impaired platelet function caused by aspirin therapy will increase the bleeding risk in the spinal surgery. Because of the risk of intraspinal bleeding and the serious outcome of subsequent epidural hematoma with associated spinal cord compression, spinal surgeons have been reluctant to operate on patients taking aspirin. For patients receiving aspirin therapy, the surgeon usually advises to discontinue the low-dose aspirin at least 7 days before the spinal surgery.^[13]^

Kang et al^[1]^ point out that discontinuation of aspirin for 7 days in aspirin-use group before spinal surgery has similar outcomes compared with the control group on operation time, estimated blood loss, postoperative blood transfusion, and bleeding risk, but may not reduce the postoperative suction drainage. But according to the present review, we find that patients undergoing spinal surgery with aspirin continuation do not have an increased bleeding risk. In addition, there was no observed increase in the operative time and postoperative blood transfusion.

Wolf et al^[14]^ report that continuation of aspirin therapy in elective pancreatic surgery such as pancreatoduodenectomy, distal pancreatectomy, and total pancreatectomy will not increase the risk of perioperative bleeding, transfusion requirement, or other major adverse complications. The study by Leyh-Bannurah et al^[15]^ shows that major surgery such as open RP and RARP can be safely performed in patients with ongoing aspirin therapy without increased blood loss. Assia et al^[16]^ also find similar results in patients undergoing routine cataract surgery. Nuttall et al^[17]^ evaluate the parameters that increase the postoperative blood transfusion in the spinal surgery, and draw a conclusion that the use of aspirin will not increase the intraoperative blood loss by the method of multiple regression analysis.

For the cardiovascular risk without aspirin continuation and mean hospital length of stay with aspirin continuation, we did not get enough samples to make an accurate decision about their relationships with aspirin. But some researches point out that the risk of perioperative cardiac events after major noncardiac surgery ranges from 1.4% among the general population older than 50 years of age and increases up to 3.9% in those who are at risk of cardiac disease.^[7]^ Aspirin decreases the risk of thrombotic events and is the most widely prescribed antiplatelet agent in clinical practice.^[18]^ A randomized controlled trial showed an absolute risk reduction of 7.2% within 30 days of major noncardiac surgery when aspirin was continued.^[19]^

Gerstein et al^[2]^ suppose that abrupt withdrawal of aspirin in the patients who have a long-term aspirin use history may induce the platelet rebound phenomenon with more TXA2 produced. Finally, major adverse irreversible cardiovascular events may happen because of the formation of thrombosis.^[2]^ Ferrari et al^[20]^ claim that stent thrombosis may be formed after aspirin discontinuation for average 10 days or as few as 4 days. They firmly believe that most of the acute coronary syndromes have the close relationships with the late stent thrombosis events due to aspirin discontinuation.^[20]^

The Brazilian Society of Cardiology guidelines for perioperative evaluation recommend that aspirin should not be discontinued in the perioperative period of vascular surgeries, as the benefit of platelet anti-aggregation outweighs the bleeding risk.^[21]^ Burger et al^[22]^ found in their meta-analysis that the discontinuation of aspirin can trigger the occurrence of cardiac, cerebrovascular, and peripheral arterial events, while the continued use of aspirin in the perioperative period of noncardiac surgeries does not increase the mortality and morbidity, despite a 50% increase in the bleeding risk.^[6]^

Gerstein et al^[2]^ indicate that physicians should make a different choice about the perioperative aspirin management when faced with different clinical conditions. Most patients who take aspirin as secondary cardiovascular prevention should continue the use of aspirin to prevent the major thromboembolic complication. For many operative procedures, including intracranial, middle ear, posterior eye, intramedullary spine, and possibly transurethral prostatectomy surgery, aspirin may be discontinued in the perioperative period to decrease the risk of perioperative bleeding.^[2]^ The influence of aspirin on cardiovascular events in spinal surgery still needs to be verified by more statistics.

There are several advantages of our study. First, we performed a detailed systematic literature review. In addition, data abstraction and quality assessment were conducted independently by 2 reviewers. These measures minimize study selection bias and allow to accurately estimate the effect of aspirin on blood loss, operative time, and postoperative blood transfusion. Second, the sample of included studies was relatively enough to evaluate the safety of continuing aspirin during spinal surgery. Third, all the 4 included articles have objectives, meeting directly to our review topic. So, the data were extracted more accurately and intact. Fourthly, the patient populations of included studies were comparable, including mainly elderly patients who required spinal surgery with the use of aspirin. Thus, we believe our findings about bleeding risk, operative time, and postoperative blood transfusion are applicable to a wide population of patients undergoing spinal surgery.

## 5 Limitations

There are several limitations of the present review that may influence the results, especially for bleeding risk. First, blood loss may have been influenced by the different anesthetic and surgical techniques. Second, the difference among cervical, thoracolumbar, and lumbar surgeries may have an effect on the total blood loss. Third, patient-related factors might influence bleeding, such as advanced age and different physical quality. Fourth, numbers of patients in aspirin group and control group were unequal in 3 included studies, which may limit comparability between groups. Fifth, dose of aspirin was slightly different in the cohort studies, which mean some patients may be at a different degree of anticoagulation before spinal surgery. This outcome may influence the bleeding events more or less.

The researches about aspirin administration in the spinal surgery can be further performed from 2 aspects. On the one hand, researches about the influence of aspirin use on cervical, thoracic, or lumbar surgery can be made separately to draw a more instructive conclusion for the need of clinical practice. On the other hand, we attempted to assess the perioperative incidence of cardiovascular events, but no enough samples reported on this outcome. The lack of consistent documentation of such data limit any conclusions in regard to cardiovascular risk. Therefore, large samples of well-designed randomized controlled trials are needed to evaluate the effect of aspirin use in spinal surgery on cardiovascular events.

With more attentions attached to the management of aspirin in the perioperative period of spinal surgery, we predict that the standard aspirin specifications will be established gradually in the future.

## 6 Conclusion

On the basis of our study, we believe that the continuation of aspirin is safe, and that the continuation of aspirin should be considered acceptable, particularly in patients who need antiplatelet therapy.
